# Residual disease after neoadjuvant chemoradiotherapy for oesophageal cancer: locations undetected by endoscopic biopsies in the preSANO trial

**DOI:** 10.1002/bjs.11760

**Published:** 2020-08-05

**Authors:** B. J. van der Wilk, B. M. Eyck, M. Doukas, M. C. W. Spaander, E. J. Schoon, K. K. Krishnadath, L. E. Oostenbrug, S. M. Lagarde, B. P. L. Wijnhoven, L. H. J. Looijenga, K. Biermann, J. J. B. van Lanschot

**Affiliations:** ^1^ Departments of Surgery Rotterdam the Netherlands; ^2^ Pathology Rotterdam the Netherlands; ^3^ Gastroenterology and Hepatology Erasmus MC University Medical Centre Rotterdam Rotterdam the Netherlands; ^4^ Departments of Gastroenterology and Hepatology, Catharina Hospital Eindhoven the Netherlands; ^5^ Amsterdam University Medical Centres – location AMC University of Amsterdam, Amsterdam Cancer Centre Amsterdam the Netherlands; ^6^ Zuyderland Medical Centre Heerlen the Netherlands; ^7^ Department of Pathology, Princess Maxima Centre for Paediatric Oncology Utrecht the Netherlands

## Abstract

**Background:**

Active surveillance has been proposed for patients with oesophageal cancer in whom there is a complete clinical response after neoadjuvant chemoradiotherapy (nCRT). However, endoscopic biopsies have limited negative predictive value in detecting residual disease. This study determined the location of residual tumour following surgery to improve surveillance and endoscopic strategies.

**Methods:**

The present study was based on patients who participated in the prospective preSANO trial with adenocarcinoma or squamous cell carcinoma of the oesophagus or oesophagogastric junction treated in four Dutch hospitals between 2013 and 2016. Resection specimens and endoscopic biopsies taken during clinical response evaluations after nCRT were reviewed by two expert gastrointestinal pathologists. The exact location of residual disease in the oesophageal wall was determined in resection specimens. Endoscopic biopsies were assessed for the presence of structures representing the submucosal layer of the oesophageal wall.

**Results:**

In total, 119 eligible patients underwent clinical response evaluations after nCRT followed by standard surgery. Residual tumour was present in endoscopic biopsies from 70 patients, confirmed on histological analysis of the resected organ. Residual tumour was present in the resection specimen from 27 of the other 49 patients, despite endoscopic biopsies being negative. Of these 27 patients, residual tumour was located in the mucosa in 18, and in the submucosa beneath tumour‐free mucosa in eight. One patient had tumour in muscle beneath tumour‐free mucosa and submucosa.

**Conclusion:**

Most residual disease after nCRT missed by endoscopic biopsies was located in the mucosa. Active surveillance could be improved by more sampling and considering submucosal biopsies.

## Graphical abstract

Remnant cancer missed by endoscopic biopsies was located in the mucosa in two‐thirds of patients with residual disease after neoadjuvant chemoradiotherapy (nCRT). One‐third of patients had residual disease in the submucosa underneath a tumour‐free mucosa. The yield of biopsies in active surveillance after nCRT could be improved by sampling larger areas of oesophageal mucosa and using biopsies targeting the submucosa.

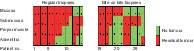

What lies beneath

## Abstract (es)

**Antecedentes:**

Se ha propuesto un seguimiento activo para los pacientes con cáncer de esófago en los que se logra una respuesta clínica completa tras quimiorradioterapia neoadyuvante (*neoadjuvant chemoradiotherapy*, nCRT). Sin embargo, las biopsias endoscópicas tienen un valor predictivo limitado para detectar la enfermedad residual. En este estudio se evaluó la localización del tumor residual tras la cirugía para poder determinar estrategias de seguimiento y endoscópicas.

**Métodos:**

Este estudio se basa en pacientes que participaron en el ensayo prospectivo preSANO (adenocarcinoma o carcinoma escamoso del esófago o unión esofagogástrica en cuatro hospitales de los Países Bajos entre 2013 y 2016). Los especímenes quirúrgicos, así como las biopsias endoscópicas efectuadas durante las evaluaciones de la respuesta clínica después de nCRT fueron revisadas por dos patólogos gastrointestinales expertos. En los especímenes de resección, se determinó la localización exacta de la enfermedad residual en la pared del esófago. Se evaluaron las biopsias endoscópicas para identificar estructuras que constituyeran la capa submucosa de la pared del esófago.

**Resultados:**

En total, 119 pacientes elegibles fueron sometidos a evaluaciones de la respuesta clínica tras nCRT seguida de cirugía estándar. Se detectó tumor residual en las biopsias endoscópicas de 70 pacientes, luego confirmadas en la histología de la pieza extirpada. Se identificó tumor residual en la pieza de resección de 27 de los otros 49 pacientes, a pesar de que las biopsias endoscópicas fueron negativas. En estos 27 pacientes, 18 presentaban tumor residual en la mucosa y ocho pacientes en la submucosa mas allá de una mucosa libre de tumor. Un paciente tenía tumor en el músculo más allá de una mucosa y submucosa libres de tumor.

**Conclusión:**

La mayoría de los casos de enfermedad residual tras nCRT que no se detectaron en las biopsias endoscópicas, se localizaban en la mucosa. El seguimiento activo podría mejorar con la toma de más muestras y considerando las biopsias submucosas.

## Introduction

After neoadjuvant chemoradiotherapy (nCRT) for locally advanced oesophageal cancer, nearly one‐third of patients have a pathologically complete response (pCR; no residual tumour cells in the resection specimen)^1^. This underlines the need to reconsider standard oesophageal resection for all patients after nCRT. Oesophagectomy is associated with postoperative mortality and high morbidity rates. Therefore, it would be beneficial if patients who continue to have a clinically complete response (cCR) during active surveillance could be spared oesophagectomy^2^. During active surveillance, frequent clinical response evaluations (CREs) are performed to assess the presence of residual locoregional disease or distant metastases. The main concern in active surveillance is residual disease remaining undetected during follow‐up. Small nests of residual disease could progress to an unresectable tumour or metastases. Accurate CREs are crucial to an active surveillance strategy.

The preSANO trial^3^, ^4^ assessed the accuracy of detecting residual disease after nCRT. Endoscopy with biopsies had a sensitivity of 69 per cent for detecting residual tumour with a tumour regression grade (TRG) of 3–4 (more than 10 per cent residual tumour cells), according to the modified Mandard score described by Chirieac and colleagues^5^. The sensitivity increased to 90 per cent when the endoscopic biopsy protocol included bite‐on‐bite biopsies to obtain tissue from the deeper layers of the oesophageal wall. Theoretically, bite‐on‐bite biopsies have the potential to reach deeper layers of the oesophageal wall and therefore to detect submucosal tumours located underneath a tumour‐free mucosa^6^. Submucosal tissue can be identified histologically by the presence of specific anatomical structures that are absent from mucosal biopsies, that is mucinous glands and thick‐walled blood vessels^7^. Although the sensitivity for detection of residual disease increased after the introduction of bite‐on‐bite biopsies, it remains unclear whether this was achieved by deeper sampling of the oesophageal wall or by the fact that, for instance, more biopsies were taken. Furthermore, biopsies alone still have a limited negative predictive value for detection of residual disease after nCRT^8^.

There is a need to investigate how endoscopic surveillance and biopsy protocols can be optimized to minimize sampling errors in this patient population. The aims of this study were to assess the exact location of undetected residual disease after nCRT and to determine the depth of bite‐on‐bite biopsies.

## Methods

The present study included patients who participated in the prospective preSANO trial^4^. All patients diagnosed with adenocarcinoma or squamous cell carcinoma of the oesophagus or oesophagogastric junction in four Dutch hospitals (2 academic hospitals and 2 high‐volume teaching hospitals) between 2013 and 2016 were screened for eligibility. Patients were considered eligible for the study if they were scheduled to undergo nCRT followed by oesophagectomy. The nCRT regimen consisted of weekly administration of carboplatin (area under the curve 2 mg per ml per min) and paclitaxel (50 mg per m^2^ body surface area) for 5 weeks concurrently with 41·4 Gy radiotherapy in 23 fractions. Patients for whom surgical resection specimens were not available for review were excluded from analysis. All patients with detected residual disease from the initiating centre (Erasmus MC – University Medical Centre) were included consecutively and comprised the control group. This group was included to gain more insight in the location of residual tumours that could be detected during CREs. Patients with undetected residual disease from all centres were defined as the study group. The study protocol was approved by the medical ethics committee of Erasmus MC (Rotterdam, MEC‐2013‐211). All patients provided written informed consent for analysis and publication. The study was registered with the Netherlands Trial Register (NTR4834).

### Baseline clinical staging and response evaluations

All patients underwent baseline clinical staging using endoscopic biopsies, endoscopic ultrasonography (EUS) with fine‐needle aspiration (FNA) of suspected relevant lymph nodes, and PET–CT. During baseline endoscopy, the distance between the incisors and upper and lower border of the primary tumour was measured. The quadrants of the oesophagus that involved tumour were specified as well. After completion of nCRT, patients underwent one or two clinical response evaluations (CREs). The first (CRE‐1) was planned 4–6 weeks after completion of nCRT, and included endoscopy with biopsies. During CREs, white‐light endoscopy was used with either regular or bite‐on‐bite biopsies using standard‐sized forceps. If no lesions were visible, at least four random biopsies were taken from the original location of the primary tumour described at baseline endoscopy. Additionally, biopsies were taken from all suspected lesions and from the borders of all ulcers. When residual vital tumour cells were detected, patients underwent PET–CT to exclude distant metastases, before oesophagectomy was performed. When no tumour cells were detected during CRE‐1, a second examination (CRE‐2) was planned 10–14 weeks after completion of nCRT. CRE‐2 consisted of PET–CT followed by endoscopic biopsies and EUS with FNA of all suspected lymph nodes. When distant metastases were detected, patients were referred for palliative care. Patients were considered to have achieved a cCR if no residual vital tumour cells were detected during CRE‐1 and CRE‐2 in endoscopic biopsies and in EUS‐guided FNA cytology. In the preSANO trial, all patients underwent standard oesophagectomy. In the present study, undetected residual disease was defined as all residual tumour with TRG 2–4 (at least 1 per cent residual tumour) in the resection specimen that was not detected during CRE‐1 and CRE‐2.

### Pathological analysis

Resection specimens and endoscopic biopsies were reviewed in all patients with residual tumour that was not detected by endoscopy during CRE (study group). The exact location of the residual tumour in the resection specimen was determined and compared with that from the control group of patients who had residual tumour detected endoscopically during CRE. Review of the resection specimens and biopsies was done independently by two experienced upper gastrointestinal pathologists. All resection specimens were processed and sampled using a standard protocol^9^. In brief, the surgical tumour bed was sampled extensively or totally. Tissue slides were stained using haematoxylin and eosin, and were subsequently evaluated to acquire information on resection margins, presence of vital tumour cells, tumour type and differentiation grade.

**Fig. 1:**
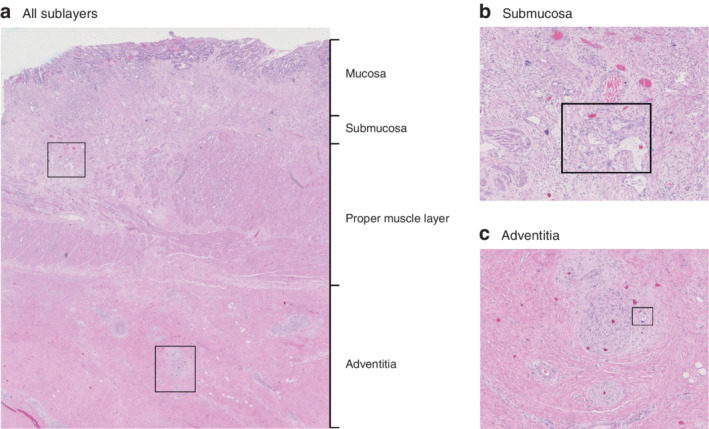
Histology of oesophageal resection specimen **a** Section from an oesophageal resection specimen showing sublayers. Detailed examples of boxed areas in the submucosa and adventitia are shown in **b** and **c** respectively. **b** The boxed area indicates glandular adenocarcinoma within an area of regressional changes in the submucosa. The submucosa was scored as tumour regression grade (TRG) 3 (more than 10 per cent vital tumour cells). **c** The boxed area shows vital tumour cells within an area of regressional changes in the adventitia. The adventitia was scored TRG 2 (10 per cent or less vital tumour cells). (Haematoxylin and eosin staining; **a** × 10 magnification, **b**,**c** × 40 magnification.)

**Fig. 2:**
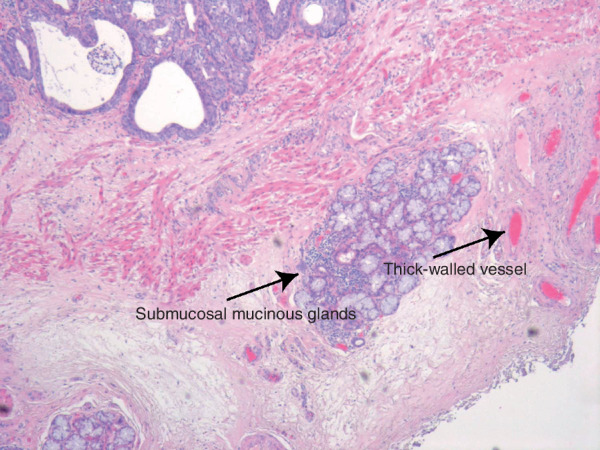
Submucosal mucinous glands and thick‐walled vesselsHistological example of a non‐irradiated (normal) area in an oesophageal resection specimen. The arrows indicate submucosal structures used to identify submucosal tissue in the endoscopic biopsies (haematoxylin and eosin staining, × 40 magnification).

Tumour cells in the resection specimen were considered vital if their cytomorphological integrity was intact. A microscopically radical resection (R0) was defined by the absence of cancer cells at the proximal, distal and circumferential margin of the resection specimen. The resection specimen was scored for overall TRG using the modified Mandard score^5^: TRG 1, no residual tumour cells; TRG 2, 1–10 per cent residual tumour cells; TRG 3, 11–50 per cent residual tumour cells; and TRG 4, more than 50 per cent residual tumour cells. The TRG was also determined for each oesophageal layer: mucosa, submucosa, proper muscle layer and adventitia. The presence of vital tumour cells was assessed relative to the area showing regressional changes (*Fig*. *1*). Further quantification of residual vital tumour cells was undertaken in all resection specimens that had undetected residual tumour cells in the mucosal layer, or in the submucosal layer underneath a tumour‐free mucosa. To evaluate the potential for detecting specific submucosal histological structures in the submucosal layer of the oesophageal wall, the relative presence of these structures (mucinous glands and thick‐walled vessels) was assessed in the non‐irradiated distal part of the oesophageal submucosa from three randomly chosen oesophageal resection specimens (*Fig*. *2*)^7^.

To gain insight into the depth of tissue sampled by endoscopic biopsies, and the potential to detect mucosal and submucosal tumours, all endoscopic biopsies taken during CRE‐1 and CRE‐2 were reviewed for both the presence of mucosal and submucosal tissue, and the presence of vital tumour cells in the submucosal tissue if applicable. The presence of submucosal tissue was defined as described above. If only mesenchymal or ulcerative tissue was detected, the nature of the tissue present in the biopsy was defined as uncertain; otherwise, the tissue was defined as mucosal.

### Statistical analysis

Descriptive statistics were used to describe baseline characteristics. Continuous variables are reported as median (i.q.r.). Student's *t* test or Mann–Whitney *U* test was used for analysis of continuous variables, and χ^2^ or Fisher's exact test for comparison of categorical data (the latter when comparing 2 categorical variables, or when events were rare). *P* < 0·050 (2‐sided) was considered statistically significant. All statistical analyses were done using the tableone package of R version 3.5.1 (R Core Team, R Foundation for Statistical Computing, Boston, Massachusetts, USA).

## Results

**Fig. 3:**
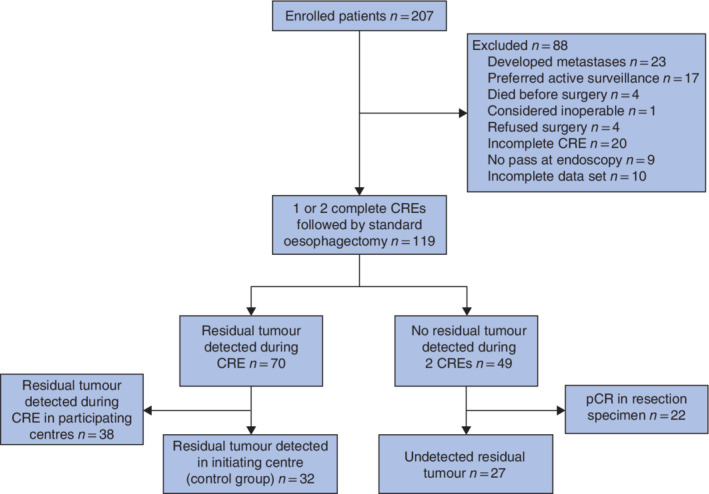
Study flow chart CRE, clinical response evaluation; pCR, pathologically complete response.

Between 2013 and 2016, 207 patients underwent nCRT, of whom 119 had one or two CREs followed by standard surgical resection. Tumour cells were detected in 70 of 119 patients during CREs, including 32 patients from the Erasmus MC – University Medical Centre who served as control group. No tumour cells were detected at CREs in 49 of 119 patients, of whom 22 had a pCR in the resection specimen. Vital tumour cells were identified in the resection specimen, which had not been detected in the endoscopic biopsies, in 27 of 49 patients (study group) (*Fig*. *3*; *Table* *S1*, supporting information). All included patients underwent CRE‐1 and CRE‐2 within a range of 28–44 and 68–91 days respectively. The bite‐on‐bite technique was used less frequently in patients with residual tumour that remained undetected. These patients also had a lower pathological T status and more often had TRG 2 residual tumour than patients in whom residual tumour was detected during CREs (control group) (*Table* *1*).

**Table 1:** Clinicopathological characteristics of patients included in analysis

	Detected residual tumour (*n* = 32)	Undetected residual tumour (*n* = 27)	*P* †
**Age (years)** *	66 (59–70)	66 (62–70)	0·937‡
**Sex ratio (M** : **F)**	28 : 4	22 : 5	0·782
**Histology**			0·447
Adenocarcinoma	25	24	
Squamous cell carcinoma	6	3	
Adenosquamous cell carcinoma	1	0	
**Preoperative T status**			0·112
cT2	2	6	
cT3	25	20	
cT4	5	1	
**Preoperative N status**			0·554
cN0	12	7	
cN1	11	9	
cN2	8	10	
cN3	1	0	
cNx	0	1	
**Type of biopsy**			0·016
Regular	6	14	
Bite on bite	26	13	
**R0 resection status**	32	27	1·000
**ypT category**			0·016
ypT1	3	11	
ypT2	8	4	
ypT3	21	12	
**ypN category**			0·079
ypN0	17	21	
ypN1	10	4	
ypN2	5	1	
ypN3	0	1	
**TRG**			0·016
TRG 2	8	16	
TRG 3	15	9	
TGR 4	9	2	

* Values are median (i.q.r.). TRG, tumour regression grade.

† χ^2^ or Fisher's exact test, except

‡ Mann–Whitney *U* test.

### Analysis of control group with detected residual disease

**Fig. 4:**
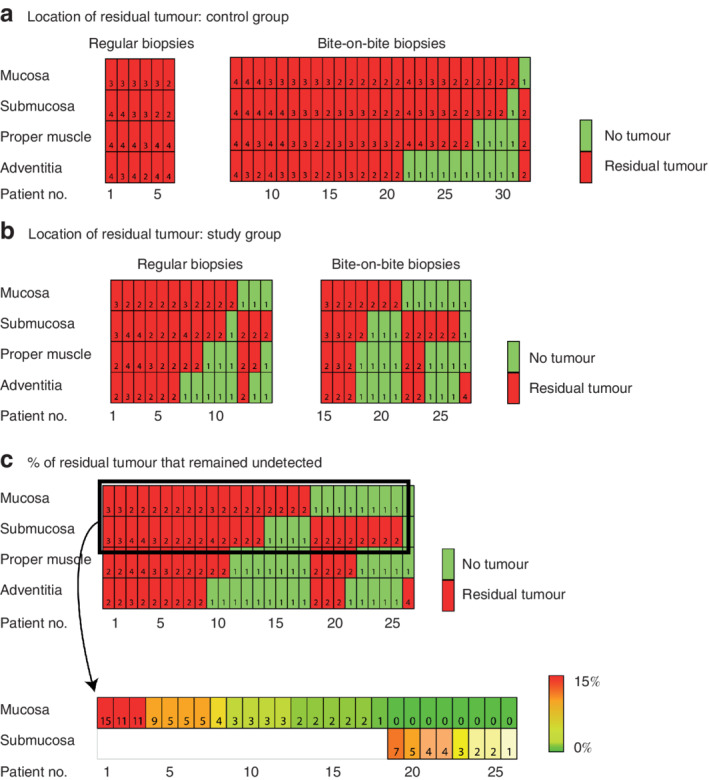
Location of residual tumours and percentage that remained undetected Location of residual tumours in **a** 32 resection specimens (control group) that were detected accurately by endoscopic biopsy after neoadjuvant chemoradiotherapy and **b** 27 resection specimens (study group) that remained undetected by endoscopic biopsy after neoadjuvant chemoradiotherapy, according to biopsy type used during clinical response evaluation (CRE). The number in each cell represents the tumour regression grade (TRG): TRG 1, no residual tumour; TRG 2–4, residual tumour. **c** Percentage of residual tumour cells present in the mucosa or submucosa that remained undetected during CRE in the study group. The results of further quantification are shown in the most superficial layer containing residual tumour cells in the mucosa or submucosa. The number in each cell in the lower part represents the percentage of vital residual tumour cells present.

Some 21 of 32 patients with detected residual disease had vital tumour cells in all layers of the oesophageal wall (*Fig*. *4a*). The mucosa and submucosa were most frequently involved; both layers were involved in 30 of 32 patients. One patient had residual disease in the submucosal layer underneath a tumour‐free mucosal layer.

For these 32 patients, tissue from endoscopic biopsies taken during 41 CREs in total were available for review (32 CRE‐1, 9 CRE‐2) (*Table* *2*). Only specific mucosal tissue was detected in the endoscopic biopsies from 16 of 41 CREs. Specific submucosal structures were detected in the endoscopic biopsies from one of the 41 CREs, using bite‐on‐bite biopsies. The origin of the tissue was uncertain in endoscopic biopsies from 24 of 41 CREs.

**Table 2:** Specific submucosal structures in endoscopic biopsies

	Detected residual tumour (32 patients)			Undetected residual tumour (27 patients)		
	All CREs (*n* = 41)	CRE‐1 (*n* = 32)	CRE‐2 (*n* = 9)	All CREs (*n* = 47)	CRE‐1 (*n* = 23)	CRE‐2 (*n* = 24)
**Submucosal structures present**						
Yes	1	1	0	3	1	2
No	16	12	4	34	17	17
Uncertain	24	19	5	10	5	5
**Type of biopsy overall**						
Regular	10	6	4	26	13	13
Bite on bite	31	26	5	21	10	11
**Type of biopsy containing submucosa**						
Regular	0			1		
Bite on bite	1			2		
**Tumour cells present in truly submucosal biopsies**	0			0		

CRE, clinical response evaluation.

### Analysis of study group with undetected residual disease

Nine of 27 patients with undetected residual disease had tumour cells involving all layers of the oesophageal wall. Residual disease was present in the mucosa in 18 patients, and in the submucosa underneath a tumour‐free mucosa in eight patients. In one patient tumour cells were present underneath tumour‐free mucosal and submucosal layers (*Fig*. *4b*). In the 26 patients with residual tumour present in the mucosa and/or submucosa, residual vital tumour cells were further quantified (*Fig*. *4c*).

The 27 patients underwent 54 CREs (*Table 2*). Of these, pathological material from endoscopic biopsies was available from 47 CREs. Specific mucosal tissue was detected in the biopsies from 34 of 47 CREs. Specific submucosal structures were identified in biopsies of three of 47 CREs from two patients, and the origin of the tissue was uncertain in ten of 47 CREs. No tumour cells were present in the biopsies that contained submucosal structures.

### Specific submucosal structures in oesophageal submucosa

**Fig. 5:**
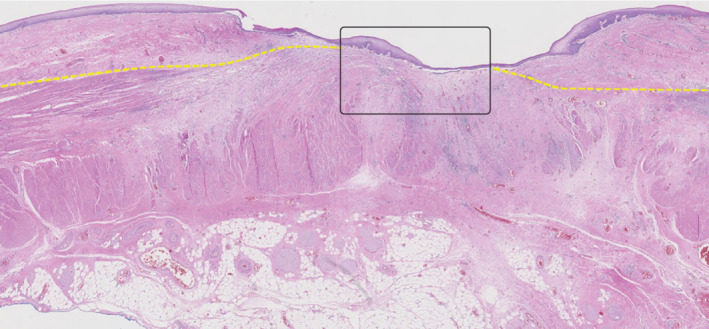
Proper muscle layer adjacent to epithelium Histological example of a resection specimen showing that the proper muscle layer, which is normally located beneath the submucosal layer, is now located adjacent to the epithelium (box), most probably owing to fibrosis after neoadjuvant chemoradiotherapy. Structures normally present in the deeper layers of the oesophageal wall have the potential to be present more superficially after neoadjuvant chemoradiotherapy. The yellow line represents the upper border of the proper muscle layer (haematoxylin and eosin staining, × 10 magnification).

In all three resection specimens, specific submucosal structures in the normal non‐irradiated oesophagus comprised 1–2 per cent of the submucosal area. Furthermore, in the irradiated part of the oesophagus, (deep) ulceration, scarring and atrophy of the subepithelial layers of the oesophagus in several instances resulted in a more superficial location of these layers than expected. *Fig*. *5* shows an example of a resection specimen in which the subepithelial tissue (lamina propria) and the submucosal tissue are fibrotic and so the upper border of the proper muscle layer lies adjacent to the epithelial surface.

## Discussion

In this study, cancer cells were still located in the oesophageal mucosa in two‐thirds of patients with residual disease after nCRT that could not be detected by endoscopic biopsies during CREs. Furthermore, nearly one‐third of patients had undetected residual disease in the submucosa underneath a tumour‐free mucosa. Whether endoscopic biopsies or bite‐on‐bite biopsies had the potential to detect these submucosal tumours is unclear, as submucosal structures were identified in only two of the 27 patients with undetected residual disease. Only one patient had undetected residual disease in deeper layers of the oesophagus beneath a tumour‐free mucosa and submucosa.

All patients included in the present study participated in a multicentre prospective trial with the objective to identify patients who might benefit from an active surveillance strategy in the future. As a result, all patients underwent standardized CREs at two fixed time points after completion of nCRT.

Undetected residual disease was found in the mucosa in two‐thirds of patients, comparable to the findings of a previous retrospective study^10^ that reported 68 per cent mucosal involvement. That study from Taiwan included solely patients with squamous cell carcinoma who had a cCR as determined by one CRE at 4–6 weeks after completion of nCRT. Unfortunately, the limited number of patients with squamous cell carcinoma in the present study makes it hard to compare squamous cell carcinoma and adenocarcinoma based on the available data. The undetected residual mucosal disease in the present study was most likely missed owing to sampling error. This could be explained by the presence of very limited and scattered residual disease in the mucosa and submucosa, which could be why endoscopic biopsies alone have shown limited negative predictive value for detection of residual disease after nCRT, both for oesophageal cancer and rectal cancer^8^, ^11^, ^12^. Sampling of larger mucosal areas, additional biomarkers or imaging is needed to decrease such sampling errors. Wide‐area transepithelial sampling (WATS) involves use of a brush (WATS^3D^®; CDx diagnostics, Suffern, New York, USA) that is able to sample larger areas of the oesophageal mucosal surface as deep as the muscularis mucosae. WATS has previously been used in an RCT^13^ for the detection of high‐grade dysplasia or adenocarcinoma in patients undergoing surveillance for Barrett's oesophagus. An absolute increase of 14 per cent in detection of high‐grade dysplasia and oesophageal adenocarcinoma was reported in a high‐risk referral Barrett's oesophagus population by using WATS compared with random endoscopic biopsies. No studies yet have reported on the use of WATS for CREs in patients with oesophageal cancer after nCRT.

Potentially valuable imaging or biomarker techniques include PET–CT with radiomics or circulating tumour DNA (ctDNA)^14^, ^15^, ^16^. Although use of PET–CT 12 weeks after completion of nCRT in the preSANO trial resulted in high false‐positive rates, its value is currently being tested in the therapeutic SANO trial beyond 12 weeks after completion of nCRT^17^. Radiomics analysis of PET–CT images (quantification of numerous imaging features) could help enhance prediction of pCR after nCRT^18^, ^19^. Use of ctDNA has shown potential in several malignancies, such as colorectal cancer, non‐small cell lung cancer and also oesophageal squamous cell cancer^20^, ^21^, ^22^, ^23^. Imaging and biomarkers could also be of value in patients who have residual disease beneath a normal mucosa and submucosa (4 per cent (1 of 27) here *versus* 9 per cent in the study of Chao *et al*.^10^) as routine endoscopic biopsies do not have the potential to reach these deeper layers.

In this study, 30 per cent of patients (8 of 27) had submucosal residual tumour below a tumour‐free mucosal layer, which is comparable to the 22 per cent reported previously^10^. Earlier studies^6^, ^24^ suggested that such tumours limited to the submucosa could be detected by bite‐on‐bite biopsies in 17–38 per cent of patients. However, most of these patients had gastric tumours and none underwent neoadjuvant therapy or had carcinoma. Therefore, these results cannot be extrapolated to the setting of oesophageal cancer after nCRT. Here, bite‐on‐bite biopsies were able to detect the cancer cells in only one of nine patients with submucosal residual disease underneath a tumour‐free mucosa. It should be noted, however, that all residual submucosal tumours underneath a tumour‐free mucosa had 10 per cent or less residual tumour (TRG 2). The preSANO trial reported that the sensitivity for detection of TRG 3–4 residual tumours increased from 69 to 90 per cent after the introduction of bite‐on‐bite biopsies. It was hypothesized that this was due to the detection of residual submucosal tumours underneath a tumour‐free mucosa. It is possible that the percentage of detected residual tumours could increase more in a surveillance setting, with endoscopic biopsies performed beyond 12 weeks after nCRT.

This study has several limitations. First, submucosal mucinous glands and thick‐walled vessels comprised only 1–2 per cent of the submucosal layer in the distal part of the non‐irradiated, normal oesophagus. Therefore, it cannot be concluded that the submucosa had not been sampled when these structures were absent from biopsies, especially if radiation‐induced atrophy and therefore the possible disappearance of these specific submucosal structures is also taken into consideration. Conversely, structures located in the deeper layers of the oesophageal wall in the healthy oesophagus could be present more superficially after nCRT owing to ulceration and fibrosis (*Fig*. *5*). As such, specific structures do not unconditionally correlate with the depth of biopsy. Second, the group of patients with undetected residual tumour was relatively small and not all resection specimens or pathological material from endoscopic biopsies were available for review. Finally, only patients with detected residual tumour from the initiating centre (Erasmus MC – University Medical Centre) were included, which could have resulted in selection bias. As the primary aim of this study was to determine the location of undetected residual tumour, additional inclusion of patients with detected residual disease would most likely not have affected the main outcomes of this study.

## Supporting information


**Table S1** ypTNM status and TRG‐status of five patients with undetected residual disease of which resection specimens were not available for revision.
