# Supplementary material for: Residual disease after neoadjuvant chemoradiotherapy for oesophageal cancer: locations undetected by endoscopic biopsies in the preSANO trial
Source: Br J Surg. 2020 Aug 5;107(13):1791–800. doi: 10.1002/bjs.11760 (PMC7689829; doi:10.1002/bjs.11760)
Supplement: Supplementary file 1 — Table S1 ypTNM status and TRG‐status of five patients with undetected residual disease of which resection specimens were not available for revision. [file BJS-107-1791-s001.docx]

**BJS11760**

**Residual disease after neoadjuvant chemoradiotherapy for oesophageal cancer: locations undetected by endoscopic biopsies in the preSANO trial**B. J. van der Wilk, B. M. Eyck, M. Doukas, M. C. W. Spaander, E. J. Schoon, K. K. Krishnadath, L. E. Oostenbrug, S. M. Lagarde, B. P. L. Wijnhoven, L. H. J. Looijenga, K. Biermann and J. J. B. van Lanschot

**Table S1 ypTNM status and TRG-status of five patients with undetected residual disease of which resection specimens were not available for revision.**

| Patient | ypTNM | TRG |
| --- | --- | --- |
| 1 | ypT1aN0 | TRG2 |
| 2 | ypT2N0 | TRG2 |
| 3 | ypT2N0 | TRG2 |
| 4 | ypT3N0 | TRG2 |
| 5 | ypT3N1 | TRG4 |
| TRG: Tumour Regression Grade | | |
